# Labyrinthine hemorrhage: practical lessons from a rare cause of acute vestibular syndrome

**DOI:** 10.1055/s-0046-1818604

**Published:** 2026-05-12

**Authors:** César Minoru Toita Koga, Marcos Christiano Lange, Valéria Cristina Scavasine, Hélio Afonso Ghizoni Teive, Henry Koiti Sato, Caio César Diniz Disserol

**Affiliations:** 1 Universidade Federal do Paraná, Complexo do Hospital de Clínicas, Departamento de Neurologia, Curitiba PR, Brazil.; 2 Instituto de Neurologia de Curitiba, Departamento de Neurologia Clínica, Curitiba PR, Brazil.; 3 Hospital Israelita Albert Einstein, Instituto do Cérebro, São Paulo SP, Brazil.

**Keywords:** Hearing Loss, Vestibular Diseases, Anticoagulants, Stroke, Hemorrhage

## Abstract

Labyrinthine hemorrhage (LH) is a rare cause of sudden sensorineural hearing loss (SNHL) and acute vestibular syndrome (AVS), which may mimic both peripheral and central etiologies. We report a case of a 76-year-old male on edoxaban therapy presenting with sudden right-sided SNHL and vertigo. Brain magnetic resonance imaging (MRI) performed 2 weeks later showed T1 and fluid-attenuated inversion recovery (FLAIR) hyperintensity of the right inner ear, consistent with LH. Evaluation of AVS in the emergency setting remains challenging, as LH may present as a rare stroke mimic, often indistinguishable from isolated labyrinthine stroke or labyrinthitis. Proper application and interpretation of vestibular assessment protocols, such as HINTS-plus, are critical in such cases. When bedside vestibular testing suggests central-type findings, but initial neuroimaging is normal, high-resolution brain MRI focused on the inner ear might be essential to rule out LH. Awareness of this rare entity may help prevent misdiagnosis, inappropriate thrombolysis, and delayed recognition, although stroke management should still be prioritized when HINTS-plus suggests a central pattern. This case highlights the diagnostic complexity of AVS and its potential pitfalls in bedside and imaging assessment, raising awareness of LH as a rare but clinically relevant stroke mimic.

## CLINICAL VIGNETTE

A 76-year-old male with a history of hypertension, dyslipidemia, and atrial fibrillation on anticoagulant therapy with edoxaban presented to an emergency department (ED) with sudden and complete right-sided sensorineural hearing loss, followed by vertigo, imbalance, nausea, and vomiting. He was treated symptomatically and discharged on oral prednisone. No structured bedside vestibular assessment or neuroimaging was performed prior to discharge. Over the following days, vestibular symptoms gradually improved, but hearing loss persisted.

Our neurological clinical outpatient evaluation after a month revealed abnormal right head impulse test and ipsilateral anacusis. Strength, reflexes, and sensory examinations were normal. No appendicular or axial ataxia was noted, and the examination of the remaining cranial nerves was unremarkable.

**Figure 1:**
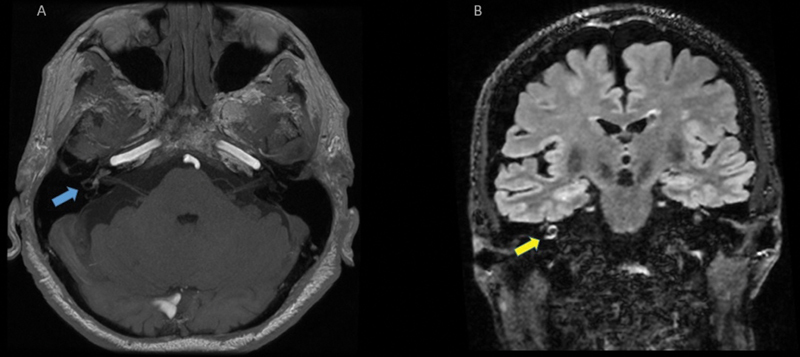
Magnetic resonance imaging (MRI) findings consistent with right labyrinthine hemorrhage. ( **A** ) Axial postcontrast T1-weighted image showing hyperintense signal in the right cochlea and vestibule (blue arrow). ( **B** ) Coronal fluid-attenuated inversion recovery (FLAIR) imaging showing hyperintensity within the right cochlea (yellow arrow).

A brain magnetic resonance imaging (MRI) taken 2 weeks after symptom onset revealed hyperintensity in the right cochlea/vestibular apparatus in T1 and in fluid-attenuated inversion recovery (FLAIR) image, suggestive of right labyrinthine hemorrhage (LH) ( Figure 1 ). Diffusion-weighted image (DWI) was normal. The patient remained asymptomatic for vestibular symptoms but had no improvement in hearing.

## FROM PRESENTATION TO RESOLUTION: LESSONS LEARNED

### What is LH and its clinical findings?

As a rare condition, characterized by bleeding within the intralabyrinthine compartment, the precise incidence and prevalence of LH remain difficult to determine due to its rarity, and it has been reported to be underrecognized. 1 Previous MRI-based studies in patients with sudden sensorineural hearing loss (SNHL) have reported a variable incidence of LH, ranging from 3.5 to 6.25%. 2 3

The earliest descriptions of LH date back to the nineteenth century, particularly in patients with leukemia. 4 Clinically, it typically presents with sudden unilateral SNHL and acute vestibular syndrome (AVS). Compared to other etiologies of sudden SNHL, hearing loss in LH tends to be more profound and affects all frequencies. 5 Very rarely, some patients may present with isolated vertigo, which has been attributed to selective bleeding confined to the vestibule, as previously reported. 6 Other clinical manifestations of LH include nausea, vomiting, vestibular nystagmus, ipsilateral lateropulsion, imbalance, and gait disturbances. 1

Although most cases are classified as idiopathic, LH has been associated with a wide range of conditions. Reported etiologies include head trauma, superficial siderosis, hematological malignancies, autoimmune diseases, viral or bacterial infections (including recent reports involving SARS-CoV-2), cerebral venous thrombosis, radiotherapy, surgical procedures, and anticoagulant therapy. In exceedingly rare cases, no identifiable risk factors are found. 1 5 7 Previously, LH was described in patients receiving anticoagulation with vitamin K antagonists; 6 however, to date, we have not find any report of an association with direct oral anticoagulants (DOACs).

### How should LH be assessed at presentation?

Patients with LH often present to the ED with AVS, a presentation that commonly triggers stroke protocols. The differential diagnosis ranges from benign, self-limited peripheral vestibular disorders to serious, incapacitating conditions, such as vestibular neuritis (VN), brainstem stroke, and isolated labyrinthine ischemic infarction (anterior inferior cerebellar artery [AICA] territory stroke), requiring prompt recognition and management. 8

An essential first step in evaluating AVS is the application of the HINTS-plus battery, designed to distinguish central from peripheral causes. This four-step bedside examination can differentiate VN from strokes in patients experiencing acute, prolonged vertigo. It comprises:

1. horizontal head impulse test (HIT);
2. evaluation of nystagmus;
3. alternate cover test for skew deviation; and
4. bedside hearing examination (finger rub).

If the HIT is normal, nystagmus is direction-changing, skew deviation is present, or asymmetric hearing loss is newly detected, a central cause such as stroke and LH should be suspected. Other findings suggest a peripheral cause, such as VN. Clinicians should be aware that the older version of the HINTS battery, which does not include a hearing assessment (HINTS without “plus”), commonly misdiagnoses labyrinthine stroke and LH symptoms as of peripheral cause. 9

The STANDING algorithm, another bedside method, may provide additional insights by incorporating assessment of equilibrium and truncal ataxia, thereby enhancing the ability to detect central etiologies. 8 Furthermore, the ABCD2 score may aid in risk stratification for stroke in AVS patients with vascular risk factors. In a large study of ED visits for AVS, stroke occurred in only 1.0% of patients with an ABCD ^2^ ≤ 3, compared to 8.1% among those scoring ≥4, and 27% in patients with scores of 6 or 7, highlighting its potential etiological relevance. 10

According to the GRACE-3 guidelines, trained emergency physicians can apply these bedside examinations with high diagnostic accuracy, often avoiding unnecessary imaging when findings are consistent with a peripheral etiology. 11 A post-test probability of stroke of up to 0.1% after a negative test in the HINTS-plus battery has been previously reported. 9 Conversely, central findings should prompt urgent neuroimaging—preferably MRI—and activation of stroke protocols, including consideration for thrombolytic therapy. 11

Unfortunately, in the present clinical case, no structured vestibular assessment was applied on symptoms onset in the ED, which led to empirical treatment with corticosteroids and delayed imaging. Proper use of bedside vestibular protocols in AVS remains a challenge to non-neurologists and might prompt earlier recognition and imaging.

### What are the main pitfalls in the initial assessment of LH?

A major concern regarding the investigation of LH is its resemblance to ischemic strokes involving the AICA. Both conditions may present with identical findings on bedside examination, 1 yet their therapeutic implications differ substantially. Table 1 summarizes key findings and the interpretation of each bedside test in AVS disorders.

**Table 1:** Findings and interpretation of bedside tests in AVS

	HIT	Nystagmus	Test of Skew	Acute hearing loss	STANDING
**Vestibular neuritis**	Abnormal (delayed to one side, with catch-up saccade)	Unidirectional	Normal	Absent	Lateralized postural instability; can walk independently
**Brainstem stroke**	Normal	Gaze-evoked/ Multidirectional	Vertical misalignment	Absent	Inability to stand or walk
**Labyrinthine ischemic infarction (AICA territory stroke)**	Abnormal (delayed to one side, with catch-up saccade)	Unidirectional	Normal	Present	Lateralized postural instability; can walk independently
**LH**	Abnormal (delayed to one side, with catch-up saccade)	Unidirectional	Normal	Present	Lateralized postural instability; can walk independently

Abbreviations: AICA, anterior inferior cerebellar artery; AVS, acute vestibular syndrome; HIT, head impulse test; LH, labyrinthine hemorrhage; STANDING, spontaneous nystagmus, direction, head impulse test, standing.

If LH is not initially suspected and remains undiagnosed in the acute phase, there is a potential risk of inappropriate thrombolysis in patients with ongoing labyrinthine bleeding. However, when central AVS is suspected, management should follow standard stroke protocols, as ischemic stroke is far more prevalent and LH remains exceedingly rare. Notably, we did not find any case of LH treated with thrombolysis in the literature.

Computed tomography (CT), which is typically employed in stroke protocols for the evaluation of sudden neurological deficits, is not effective in detecting disorders affecting primarily the membranous labyrinth, such as intralabyrinthine hemorrhage or inflammatory disorders. These conditions can only be reliably visualized with MRI. 2 6 While clinical neurological examination is known to outperform this exam in distinguishing stroke from VN, particularly because most cases of labyrinthine ischemia present with normal MRI findings, 9 12 this does not apply to LH. 1 When carefully analyzed, brain MRI can identify intralabyrinthine hemorrhage in the acute phase, especially when performed on 3T systems using thin-section sequences targeting the vestibular apparatus. Characteristic findings include T1- and FLAIR-hyperintensities. 6 Thus, MRI may assist in differentiating LH from labyrinthine ischemia and VN, as outlined in Table 2 .

**Table 2:** Differences between VN, labyrinthine ischemia, and LH in MRI imaging

	T1	FLAIR	DWI
**VN**	Normal	Normal / Hyperintense	Normal
**Labyrinthine ischemic infarction (AICA territory stroke)**	Normal	Normal	Generally normal Very rarely labyrinthine or vestibulocochlear nerve infarction
**LH**	Hyperintense	Hyperintense	Normal

Abbreviations: AICA, anterior inferior cerebellar artery; DWI, diffusion-weighted image; FLAIR, fluid-attenuated inversion recovery; LH, labyrinthine hemorrhage; MRI, magnetic resonance imaging; VN, vestibular neuritis;

However, it is important to note that high signal intensity on T1- and FLAIR-weighted MRI sequences is primarily associated with the presence of methemoglobin and increased protein concentration within the labyrinthine fluids—features that tend to become apparent between 3 and 14 days after hemorrhage onset. Therefore, MRI performed in the hyperacute phase must be interpreted with caution.

One recent study reported a case of LH missed on MRI performed within 24 hours of symptom onset. 13 Moreover, early MRI can also miss infarctions in the posterior fossa. Approximately 15 to 20% of ischemic strokes in this region may not be detectable within the first 24 hours of symptom onset. Similarly, labyrinthine ischemia is often not visualized on early imaging. 12 Delayed high-resolution MRI, with a focus on the internal auditory canal and vestibular structures, may improve diagnostic accuracy—albeit at the cost of losing the window for early ischemic stroke management.

Taken together, these considerations highlight several practical points. First, when bedside findings suggest central involvement, clinicians should initiate stroke protocols and obtain urgent neuroimaging. Even if initial imaging is negative, LH is rare, and its possibility should not delay appropriate acute stroke treatment, including thrombolysis.

To minimize inappropriate thrombolysis, clinicians might rapidly assess stroke risk (ABCD ^2^ score) alongside bleeding risk - considering bleeding history, family history of bleeding disorders, hepatic or renal dysfunction, and details of previous procedures or anticoagulation-related events – although no validated tool exists for this specific scenario. 14 If the patient is outside the therapeutic window for acute stroke management, a high-resolution MRI after 24 to 72 hours from symptom onset is recommended. Conversely, when bedside testing indicates a peripheral pattern and clinical risk is low, routine MRI is often unnecessary. 9

### What are the main treatment options for LH, and what is the expected prognosis?

There is a lack of high-quality evidence to guide LH treatment, given its rarity. Clinical manifestations like SNHL may be managed with corticosteroids, while vertigo is addressed symptomatically. 1 In cases where LH is associated with anticoagulant therapy, we propose the following approaches:

- Consider resuming anticoagulation 30 days after symptom onset in high-risk patients, as for intracerebral hemorrhage;
- Consider switching to an anticoagulant with a lower bleeding risk, such as apixaban, as suggested in previous trials. 15

The prognosis of hearing impairment in LH is generally poorer than that associated with other etiologies of sudden SNHL. Most patients experience irreversible hearing impairment. In contrast, vestibular symptoms typically resolve within a few weeks, owing to central compensation between bilateral vestibular systems. 5

In conclusion, as a rare cause of sudden-onset SNHL and AVS, LH almost invariably results in permanent ipsilateral deafness. Bedside evaluation of AVS etiologies remains challenging, as LH can mimic both peripheral and central disorders, leading to potential diagnostic and therapeutic pitfalls. Careful application and interpretation of vestibular assessment protocols such as HINTS-plus is essential in these cases.

When HINTS-plus suggests a central pattern but neuroimaging is initially negative, repeat or high-resolution MRI focused on the inner ear may be warranted to rule out LH or subtle posterior fossa infarcts. Nonetheless, LH is an uncommon condition, and its consideration should not delay stroke-specific management, including thrombolysis when appropriate—common conditions should still be prioritized in acute decision-making. Given the absence of evidence-based treatment guidelines, management of LH should be individualized.

Ultimately, this case underscores the diagnostic complexity of AVS, highlights potential pitfalls in bedside and imaging assessment, and raises awareness of LH as a rare but clinically relevant stroke mimic.

## Notes

TEACHING POINTS

1. Labyrinthine hemorrhage (LH) is a rare cause of sudden-onset sensorineural hearing loss and acute vestibular syndrome (AVS), often underrecognized in emergency settings.
2. There are similarities between LH and isolated labyrinthine stroke or labyrinthitis, with HINTS-plus suggesting a central cause. High-resolution magnetic resonance imaging (MRI) plays a central role when LH is suspected.
3. Clinicians should manage suspected central AVS following standard stroke protocols, yet remain aware that LH, though exceedingly rare, can mimic stroke.
